# Patient preferences for reducing bowel adverse events following prostate radiotherapy

**DOI:** 10.1371/journal.pone.0235616

**Published:** 2020-07-08

**Authors:** Mark V. Mishra, Winter Maxwell Thayer, Ellen Janssen, Bradford Hoppe, Caitlin Eggleston, John F. P. Bridges

**Affiliations:** 1 Department of Radiation Oncology, School of Medicine, University of Maryland, Baltimore, MD, United States of America; 2 Bloomberg School of Public Health, Johns Hopkins University, Baltimore, MD, United States of America; 3 Johns Hopkins University School of Nursing, Baltimore, MD, United States of America; 4 Center for Medical Technology Policy, Baltimore, MD, United States of America; 5 Department of Radiation Oncology, Mayo Clinic-Florida, Tampa, FL, United States of America; 6 Department of Biomedical Informatics, The Ohio State University, Columbus, OH, United States of America; University of Pennsylvania, UNITED STATES

## Abstract

**Background:**

The Extended Prostate Cancer Index Composite (EPIC) instrument is a commonly used patient reported outcome (PRO) tool in prostate cancer clinical trials. Summary scores for EPIC subscales are calculated by averaging patient scores for attributes (e.g., side effects), implying equal weighting of the attributes in the absence of evidence showing otherwise.

**Methods:**

We estimated patient preferences for each of the attributes included in the bowel subscale of the EPIC instrument using best-worst (B-W) scaling among a cohort of men with prostate cancer. Patients were presented with multiple tasks in which they were asked to indicate which attribute they would find most and least bothersome at different levels of severity. Analysis utilized both (simple) B-W counts and scores to estimate patient preferences for each attribute as well as attribute levels.

**Results:**

A total of 174 respondents from two institutions participated in the survey. Preference estimates for each of the five attributes included in the EPIC-26 bowel subscale showed wide variation preferences: ‘losing control of bowel movements’ was found to be the most bothersome attribute, with a B-W score of -0.48, followed by bowel urgency which also had negative B-W score (-0.04). Increased frequency of bowel movements was the least bothersome attribute, with a B-W score of +0.33, followed by bloody stools (+0.12), and pelvic/rectal pain (+0.06). Analysis of preference weights for attribute bother levels showed preference estimates be linear.

**Conclusions:**

We provide novel evidence on patient preferences for side effect reduction following prostate radiotherapy. Within the bowel sub-scale of the EPIC-26 short form, we found that bowel incontinence was perceived to be the most bothersome treatment effect, while increased bowel frequency was least bothersome to patients.

## Introduction

It is estimated that there will be nearly 175,000 new cases of prostate cancer diagnosed in the US in annually [1]. With the widespread use of PSA screening [2], the majority of men are diagnosed with localized prostate cancer and choose to receive treatment with surgery or radiation. Both treatment approaches result in similar—and high—rates of survival [3], but are associated with a distinct toxicity profile, making patient preferences related to quality-of -life a central part of the treatment decision-making process [4, 5].

Radical prostatectomy is associated with higher rates of urinary incontinence and sexual dysfunction, while radiation therapy results in more urinary obstructive symptoms and bowel effects [5]. For patients who choose to receive RT over surgery, they must also choose between multiple different RT modalities: intensity-modulated radiation therapy, brachytherapy, proton beam therapy, and stereotactic body radiation therapy [6]. Although each of these approaches has established efficacy, the lack of superiority of one approach over the other in terms of survival or quality-of-life, makes treatment decision-making a difficult process for patients. Comparative effectiveness studies are underway to better document patient-reported outcomes (PROs) following treatment with each of the different radiation modalities [7]. A pivotal randomized study comparing protons vs. photons for men with low- and intermediate-risk prostate cancer is currently being conducted, and the primary objective of study is to compare PROs related to bowel function [7].

Current methods for evaluating PROs typically involve averaging patient responses across the different attributes (e.g., side effects) measured in PRO instrument [8]. For example, in the bowel subscale of the expanded prostate cancer index tool—one of the most commonly used PRO tools used in prostate radiotherapy trials—patients are asked to rate the severity of their symptoms across 5 different side effects: bowel frequency, bowel urgency, incontinence, bloody stools, and pain. Summary scores are calculated by averaging patients across each of these 5 attributes. Although this provides a conceptual framework for understanding the composite impact of different treatment options, such analyses are based on the assumption that: 1) each side effect is of equal significance to patients, and 2) the levels of severity of the side effects can be measured on a linear scale [9]. An understanding of patient preferences for different health states measured in PRO instruments will be important to consider in the context of comparative effectiveness studies.

Best practices guidelines for scientifically studying and quantifying patient preferences through stated preference methods have now been published [10–12]. In oncology, these methods have been utilized to successfully evaluate and quantify patient preferences related to different chemotherapy and surgical toxicities [13–15]. However, there is a paucity of literature focused on patient preferences related to different radiation-induced side effects. Here, we utilized stated-preference techniques to determine which bowel side effects prostate cancer patients find to be most impactful.

## Materials and methods

Best-worst (B-W) scaling (case 2), also known as the profile case, was used to elicit preferences across bowel side-effects of prostate cancer treatment. B-W case 2 presents participants with a series of choice tasks where each attribute is shown with varying levels and the most and least preferred options are indicated [12, 16]. Analytic efficiency is maximized in B-W scaling because in each task information is collected about the attribute and level selected as best and worst, as well as those that are not selected. B-W case 2 has the advantage over other types of B-W scaling (e.g., B-W case 1 or ‘object case’) in that information is collected on levels of each attribute in addition to the attributes themselves.

The attributes and levels in this study were adapted from the Extended Prostate Cancer Index Composite Short Form (EPIC-26) [17]. The EPIC-26 contains five domains (urinary incontinence, urinary irritative/obstructive, bowel, sexual, and hormonal). Subscale scores are calculated by linearly transforming Likert ratings to a scale that ranges from 0 to 100 where higher scores indicate higher health-related quality of life. For example, the bowel subscale asks participants to rate how big a problem they have had with each item, and with bowel habits overall over the past four weeks on a five-point Likert scale (0 = no problem, 1 = very small problem, 2 = small problem, 3 = moderate problem, 4 = big problem). The rating of each item is multiplied by 25; then all items in the subscale are averaged to create a subscale score.

**Fig 1:**
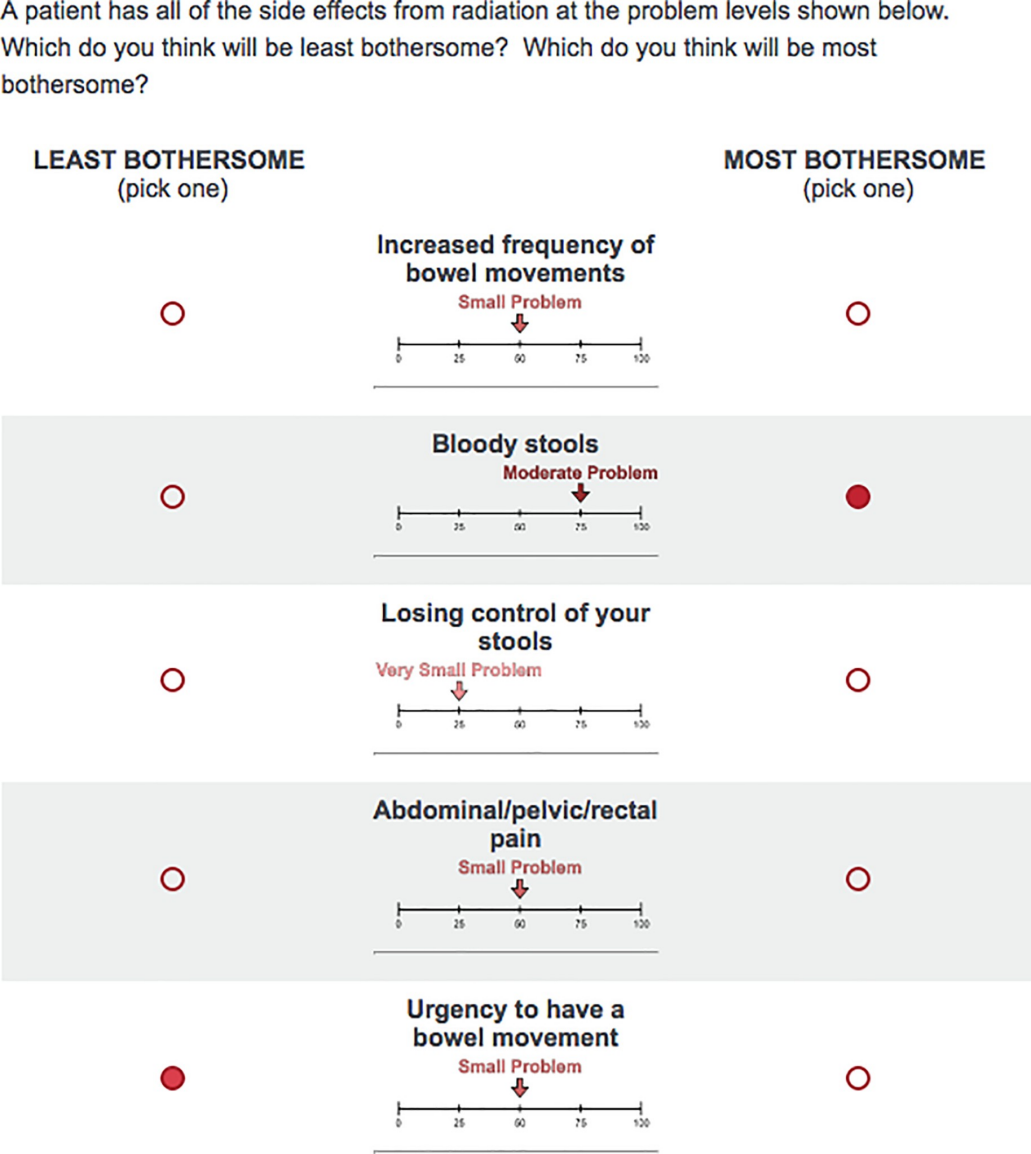
An example of a task as shown to participants.

We derived the attributes for this study from the bowel subscale, without the overall rating, providing five attributes. The middle of the Likert scale was retained so that each attribute had three possible levels (very small, small, or moderate). These levels were chosen as these levels represent the majority of bother levels that are reported by patients based on published data using the EPIC questionnaire in modern radiation therapy clinical trials. We framed each task with a short vignette that asked participants to indicate which side-effect they would find most and least bothersome (Fig 1). The complete IRB approved version of the survey is included as a S1 Appendix.

The experimental design of a stated preference study refers to the particular combination of attributes and levels over the task set that participants evaluate [12]. The design needs to have statistical properties that allow for estimation of preference weights. Our design was balanced and orthogonal with 18 tasks, meaning that levels of each attribute appeared the same number of times (6) and varied independently across tasks.

The survey was pre-tested with a small group of prostate cancer patients (n = 10) from University of Maryland School of Medicine. All attributes and levels were retained.

### Sample

Following approval of this study by the Intuitional Review Boards of the University of Maryland, Baltimore and University of Florida, participants were recruited via email from the University of Maryland Medical Center and in-clinic at the University of Florida. Patient recruitment was between June- September 2017. A waiver of documentation of consent was approved by the IRB. The following language was included at the start of the survey: *By deciding to complete the survey*, *each participant will be involved in the research necessary for this survey*. *Participation is voluntary*. *Refusal to participate will involve no penalty or loss of benefits to which the subject is otherwise entitled*, *and the subject may discontinue participation at any time without penalty or loss of benefits to which the subject is otherwise entitled*.

**Fig 2:**
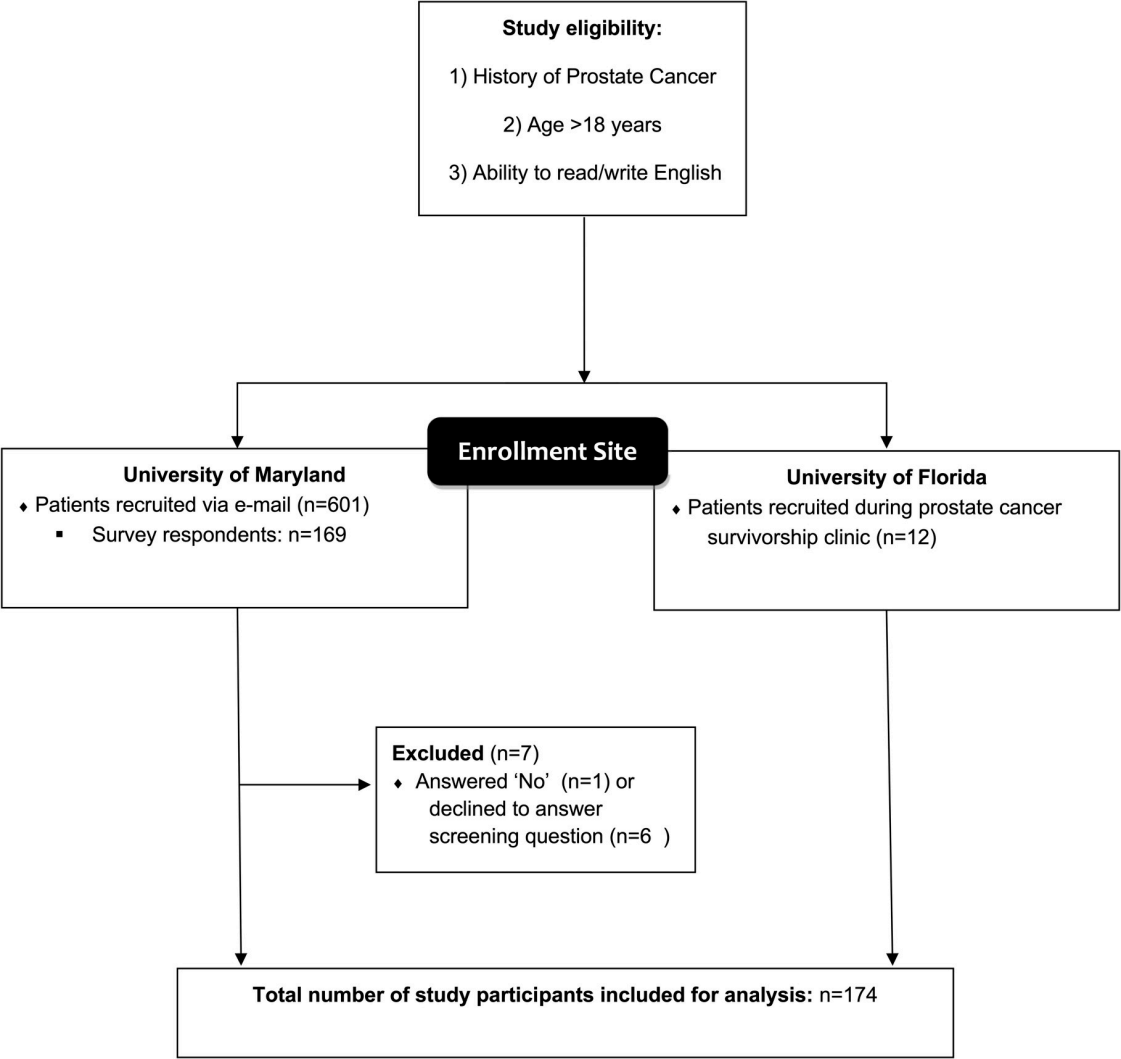
CONSORT diagram.

Responses were collected electronically at the University of Maryland, whereas surveys from the University of Florida were performed on paper due to limited computer access within the clinic. Participants who completed the survey electronically were randomized to see a subset of 9 out of 18 tasks using block randomization so that each block has the same properties as the original survey design (e.g., orthogonal design with the levels of each attribute appearing the same number of times varying independently across tasks). The electronic version of the surveys was conducted using Qualtrics^XM^ (Utah, USA). Participants who participated in the paper-based surveys completed all 18 tasks, as block randomization maintaining survey design properties was not possible when conducting paper-based surveys. To be eligible, participants must have had a previous diagnosis of prostate cancer, be at least 18 year old, and have the ability to read/write in English. Patients were offered an optional $15 gift card to recognize their time for participating in the survey. A CONSORT diagram is shown in Fig 2.

### Data analysis

The number of times each attribute level was chosen as best (i.e., ‘least bothersome’) and worst (i.e., ‘most bothersome’) was summed across all tasks. B-W scores for each attribute level were then estimated as the difference between the number of times each attribute level was selected as ‘best’ from the number of times it was selected as ‘worst’, divided by the total number of appearances of the attribute level. Missing responses were not counted in the denominator of the B-W score.

We then estimated the preference (bother) estimates of the individual attributes by estimating B-W scores for each of the 5 attributes included in the bowel sub-scale of the EPIC-26 tool. B-W scores for each attribute were calculated by subtracting the number of times each attribute was chosen as ‘best’ (e.g., ‘least bothersome’) from the number of times it was chosen as ‘worst’ (e.g ‘most bothersome’) at any level, and then divided the result by the number of appearances of the attribute in the survey.

Mean bother scores for each attribute were then calculated on a bother scale ranging from 0 to 100; an attribute being chosen (at any level) as ‘most bothersome was given a score of ‘100,’ and an attribute being chosen as ‘least bothersome was given a score of ‘0.’ Attributes that were not selected as ‘most’ or ‘least’ bothersome, were given a score of ‘50.’ To calculate the relative preference/bother weight of the 5 attributes included in the EPIC-26 subscale, the mean bother score for each attribute was divided by the sum of the mean scores for all 5 attributes.

After bother estimates for the individual attribute were assessed, bother estimates for the different levels of each attribute were evaluated by estimating the relative attribute level importance. Relative attribute level importance was calculated by estimating the amount of variability within each attribute divided by the amount of variability across all attributes. That is, the difference between the lowest level B-W score and the highest level B-W score (maximum score—minimum score) was calculated for each attribute; this difference was then summed across all attributes. For each attribute, the difference was divided by the sum of all the differences.

## Results

A total of 181 respondents provided data. Seven respondents answered no (1) or did not answer (6) the screening question (have you been diagnosed with prostate cancer?), and were excluded from analysis. An additional 41 participants did not complete 50% or more of the B-W tasks. The mean age (SD) of respondents was 68.6 (7.54) years, and the majority of respondents (n = 105, 60%) were non-Hispanic white. Eighty-one (46%) patients had previously undergone external beam radiation therapy with photons and 51 (29%) had received proton beam therapy. See Table 1 for participant characteristics.

**Table 1:** Patient characteristics.

Characteristic	Total (n = 174)
**Age**	68.61(7.54)
**Ethnicity**	
Non-Hispanic White	105
Black or African American	14
Do not wish to identify	4
Hispanic/Latino	1
Unknown	50
**Marital status**	
Single, never married	9
Married or domestic partnership	100
Widowed	5
Divorced	10
Unknown	50
**Education**	
No High School	3
High School or GED	11
College or technical school	62
Graduate School	48
Unknown	50
**Diagnosis year**	
Before 2013	31
2013 to 2014	21
2015 or after	103
**Risk Group**	
Not sure	13
Low-risk	25
Intermediate-risk	53
High-risk	61
Metastatic	3
**Treatment type**	
External beam radiation therapy	81
Androgen deprivation therapy (hormone therapy)	53
Proton beam therapy	51
Prostatectomy (surgery)	27
Prostate brachytherapy	24
Active surveillance	15

Best-worst counts as well as mean B-W scores for attribute and attribute levels, along with relevant measures of variability and uncertainty, are presented in Table 2. A positive score indicates that a level was chosen as ‘least bothersome’ more often than ‘most bothersome,’ and a negative score indicates a level was chosen as ‘most bothersome’ frequently than ‘least bothersome’. ‘Losing control of your stools’ had the lowest scores (e.g., chosen as ‘most bothersome more frequently): very small problem = -0.31, small problem = -0.45, moderate problem = -0.67. ‘Frequency of bowel movements’ had the highest scores (e.g., chosen as ‘least bothersome’ more frequently: very small problem = 0.54, small problem = 0.32, moderate problem = 0.14.

**Table 2:** Best worst counts and scores for attribute levels and relative attribute level importance.

Attribute	Levels	Best	Worst	Appearances	Mean Best-Worst	SEs	Relative attribute level importance
Urgency	Moderate	31	129	435	-0.23	0.027	19%
	Small	46	65	424	-0.05	0.025	
	Very small	112	47	428	0.15	0.029	
	Total for all levels	189	241	1287	-0.04	0.016	
Pain	Moderate	39	98	429	-0.14	0.026	21%
	Small	57	32	410	0.06	0.023	
	Very small	139	22	448	0.26	0.026	
	Total for all levels	235	152	1287	0.06	0.015	
Control	Moderate	10	290	415	-0.68	0.025	19%
	Small	17	215	437	-0.45	0.027	
	Very small	32	168	435	-0.31	0.029	
	Total for all levels	59	673	1287	-0.48	0.016	
Bloody stools	Moderate	52	82	433	-0.07	0.027	21%
	Small	76	33	430	0.10	0.024	
	Very small	170	26	424	0.34	0.029	
	Total for all levels	298	141	1287	0.12	0.016	
Frequency	Moderate	106	45	431	0.14	0.028	20%
	Small	150	16	425	0.32	0.026	
	Very small	250	19	431	0.54	0.028	
	Total for all levels	506	80	1287	0.33	0.016	

**Fig 3:**
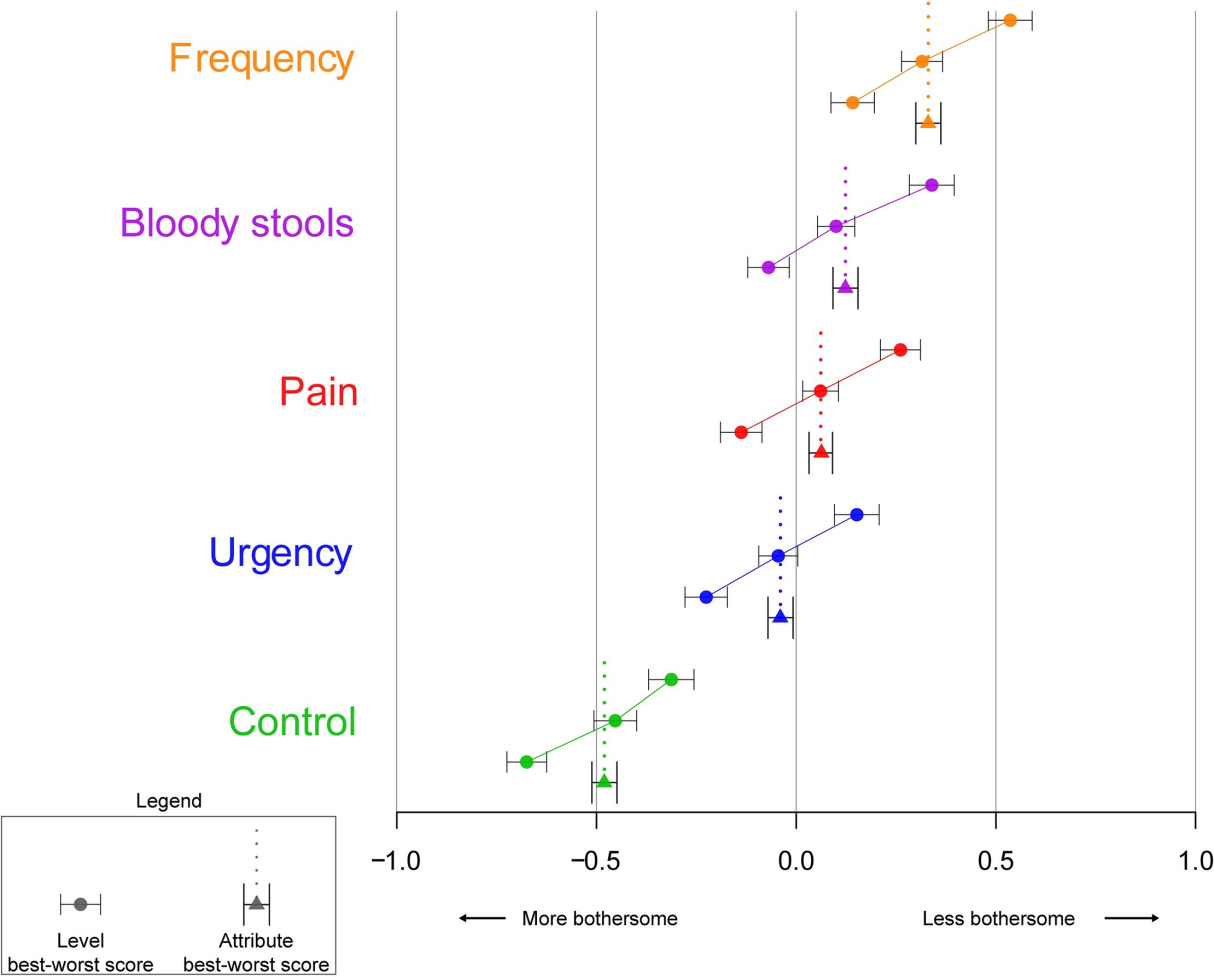
Best worst counts and scores for attribute levels and attributes.

When assessing the bother estimates of the five attributes included in the EPIC-26 bowel subscale, losing control of bowel movements was found to be the most bothersome attribute, with a B-W score of -0.48. Bowel urgency also had a negative B-W score (-0.04). Increased frequency of bowel movements was the least bothersome attribute, with a B-W score of 0.33, followed by bloody stools (0.12), and pelvic/rectal pain (0.06) (Table 2). The results are also displayed in Fig 3.

**Fig 4:**
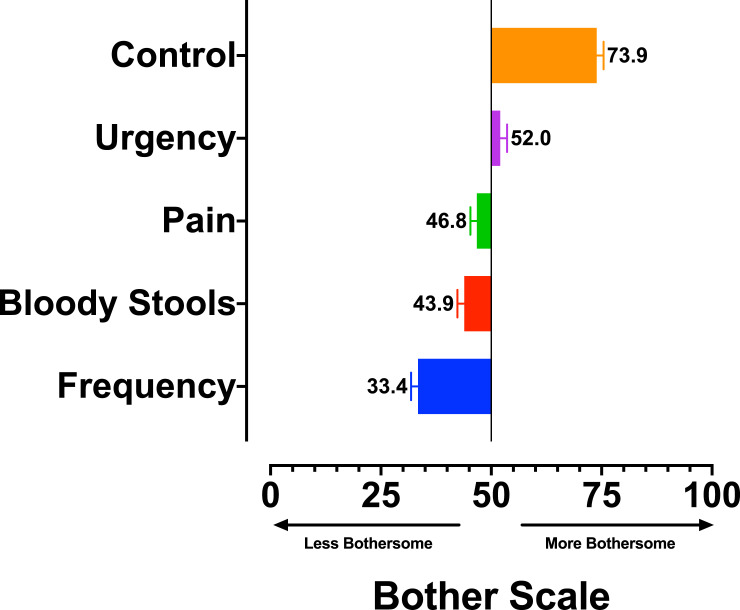
Attribute bother scores.

**Fig 5:**
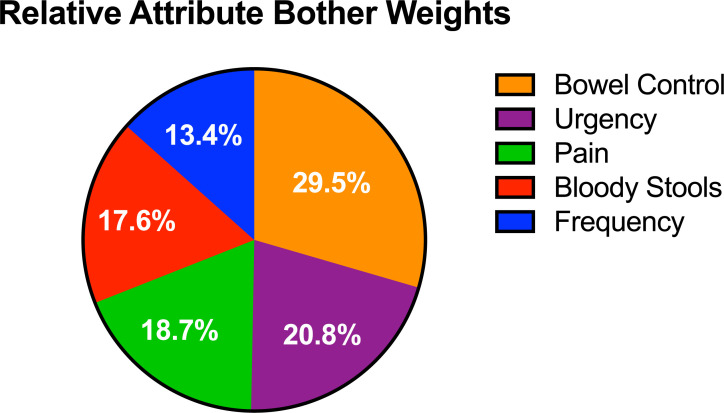
Proposed relative bother weights for attributes.

Mean attribute bother scores were calculated on a scale of 0–100, with 100 corresponding to ‘most bothersome,’ 0 corresponding to ‘least bothersome,’ and a score of 50 corresponding to a neutral bother score. Mean scores are shown in Fig 4. Relative bother weights for the 5 attributes were calculated based off the mean bother scores for all attributes. Each of the 5 attributes in the EPIC-26 score have equal weighting (20% each). However, the relative bother weights for the attributes based on the B-W scores, ranged from 29.5% (losing control of stools) to 13.4% (bowel frequency). Relative bother weights for each of the 5 attributes are shown in Fig 5.

When evaluating the bother estimates across levels of each attribute, the relative attribute level importance was approximately equal across all attributes, indicating that the levels can be scored on a linear scare. Attribute level importance ranged from 21% for ‘abdominal/rectal/pelvic pain,’ to 19% for ‘losing control of your stools’ (Table 2).

## Discussion

An understanding of patient preferences for prostate cancer is particularly important, given that patients have multiple different treatment options that are equivalent in terms of efficacy, but result in different side effect profiles [5, 18, 19]. A large randomized study has recently shown that use of a preference-based tool to help patients choose between surgery, radiation, or active surveillance is associated with improved patient satisfaction with care [20]. Our study was focused on patient preferences specific to the patient reported outcome measures (‘attributes’) included in the bowel subscale of the EPIC-26 short-form, one of the most common PRO instruments used in prostate cancer clinical trials, and the PRO tool that is being used to assess the primary endpoint of the ongoing proton vs. photon randomized study for prostate cancer [7]. The patient preferences observed in this study challenge the scoring methodology currently used in the EPIC questionnaire.

Our study found a wide variation in preference each of the 5 attributes included in the bowel sub-scale. For example, ‘Losing control of stools,’ an attribute included in the PRO to measure bowel continence, was found to be the attribute that patients found most bothersome regardless of the bother level of this attribute. Conversely, bowel frequency was found to be attribute that patients found to be least bothersome. The remaining three attributes (bloody stools, pelvic/rectal pain, and urgency) were found to be roughly equal in terms of preference/bother estimates. The bowel sub-scale currently weights these five attributes equally (20% each). However, our analysis found that the bother weight for ‘losing control of stools’ to be more than twice as high of that of bowel frequency (0.295 compared to 0.134). If this finding is validated in future studies, studies using the EPIC-26 PRO should consider reporting both un-weighted and weighted scores.

Attribute levels (e.g., different levels of bother) for measures in EPIC-26 are currently scored using a linear scoring system. Individual attributes are scored using a 5-point Likert scale: 0 = no problem, 1 = very small problem, 2 = small problem, 3 = moderate problem, 4 = big problem. Previous studies have shown that patient preferences for different levels within and across attributes measured in quality-of-life tools to be non-linear in oncology [21]. For example, a conjoint analysis used to assess patient preferences for different health states measured in the EORTC QLQ-C30 study showed strong non-linearities within each of the domain measures of the EORTC QLQ-30. However, in this study, we found preference estimates for the different levels of each attribute included in the bowel sub-scale of EPIC-26 to be linear, which supports the current scoring system for attribute levels in EPIC-26 bowel sub-scale. It is important to note that our study only measured preferences for three item levels (very small, small, or moderate problems) for each attribute, given that these are the more commonly experienced attribute levels reported by patients undergoing prostate radiotherapy. Therefore, it is possible that non-linearities exist between moderate to big problem, but this was not evaluated in the current study.

The results of this study have relevance to several of the ongoing efforts to establish the comparative effectiveness of alternative radiation modalities used to treat prostate cancer. For example, a comparison of PROs using the EPIC-26 tool for patients treated with protons vs. photons found no differences between bowel, summary scores between the two cohorts. However, when comparing the responses for each of the attributes, significant differences were found for rectal urgency and frequent bowel movements [19]. Similarly, a comparison of PROs for men with prostate cancer treated with IMRT vs. 3D-CRT found no differences between the two cohorts when only comparing PRO summary scores [22]. The results of our study raises the questions of whether significant differences would have been found if a preference-weighted comparison of PROs was performed. While the results of our analysis cannot be directly applied to these analyses, they do highlight the importance of considering preference-weighted analyses when comparing PRO measures in comparative effectiveness studies. The importance of evaluating patient preferences in health technology assessments has been highlighted by recent inclusion of data related to patient preferences in regulatory decisions by the U.S. Food and Drug Administration.

Another novel finding from our study is the importance of evaluating individual attribute preference weights in addition to preference weights for attribute levels when performing a B-W case 2 analyses. Preference results can be confounded by the fact that it is impossible to separate the effect of an attribute from the effect of its levels [23]. For example, if a participant chooses a very small increase in frequency of bowel movements as the best aspect of a profile, we cannot isolate the effect of the attribute (frequency of bowel movement), the level (very small), or the combination of the two. Therefore, results may be due to scale differences within the levels of each attribute, or to differences in the preference weight of the attributes. Previously published B-W case 2 studies have focused on the attribute level weights; however, our study found significant differences in the overall attribute preferences, but not between the levels of the attributes. We recommend future B-W case 2 analyses report overall attribute preference weights in addition to attribute level preference weights as this data is complimentary.

There are several limitations of the present study that are worth noting. First, the majority of patients who participated in our study had already been through cancer treatment. Patients may have had some level of subjective bias for the different attributes based upon their own treatment experience. Second, we only evaluated patient preferences for the bowel sub-scale of the EPIC-26 instrument. However, patients undergoing prostate radiotherapy may also experience urinary symptoms or sexual dysfunction. Future studies will be necessary to elicit patient preferences within and across all three subscales included in the EPIC instrument. Reporting of patient demographics, tumor and treatment characteristics was voluntary and via patient-self report, resulting in a large number of patients who did not report this information. The majority of men who did report their race were White, which may have biased our results. Future studies with large patient cohorts with robust sociodemographic and clinical data will be necessary to evaluate preference heterogeneity within different subgroups of the patient population. Finally, a small number of participants in our study (n = 12/174) completed a paper survey rather than an electronic (e.g., computer-based) survey that had 18 tasks rather than a block of 9 randomized tasks in the computer-based survey. This could have contributed to measurement error if participants completing the full set of tasks became fatigued over time.

## Conclusion

We provide novel evidence on patients’ preferences for side effect reduction following prostate radiotherapy. The use a preference-weighted score may provide a more accurate reflection of patient-perceived quality-of-life impact following treatment, and may ultimately help facilitate shared-decision making between prostate cancer patients and clinicians. Future research is are needed to validate these findings in a larger patient cohort and across a wider range of attributes.

## Supporting information

S1 Appendix (DOCX)
