# Supplementary material for: Patient preferences for reducing bowel adverse events following prostate radiotherapy
Source: PLoS One. 2020 Jul 8;15(7):e0235616. doi: 10.1371/journal.pone.0235616 (PMC7343167; doi:10.1371/journal.pone.0235616)
Supplement: S1 Appendix — (DOCX) [file pone.0235616.s001.docx]

For each of the following sets of side effects, we would like you to think about how a friend who has been diagnosed with prostate cancer might feel. Please tell us which side effect you think *your friend* would consider the **most bothersome** and which one *your friend* would consider the **least bothersome**. Even if you think that all of them would be bothersome, or that none of them would be bothersome, please choose the most and least bothersome side effect.

**Example Question**

For example, James is taking the survey. James is asked to consider a friend that has been diagnosed with prostate cancer. His friend faces a treatment that has the following side effects. James chooses which side effect he thinks his friend will find most bothersome and which side effect his friend will find least bothersome.

| **Side effect** | **Problem size** | **Most Bothersome (Pick one)** | **Least Bothersome(Pick one)** |
| --- | --- | --- | --- |
| Increased frequency of bowel movements | big problem | 🞏 | 🞏 |
| Bloody stools | moderate problem | 🞏 | ◼ |
| Losing control over your stools | big problem | 🞏 | 🞏 |
| Abdominal/pelvic/rectal pain | small problem | 🞏 | 🞏 |
| Urgency to have a bowel movement | small problem | ◼ | 🞏 |
|  |  |  |  |

It was difficult to choose, but James picked that his friend would think that the most bothersome side effect is a small problem with urgency to have a bowel movement and that the least bothersome side effect is a moderate problem with bloody stools.

Now it is your turn to answer this type of question. You will see 18 questions like this.

1. Please select the side effect that you think your friend will find most bothersome and the side effect your friend will find least bothersome.

| **Side effect** | **Problem size** | **Most Bothersome (Pick one)** | **Least Bothersome(Pick one)** |
| --- | --- | --- | --- |
| Increased frequency of bowel movements | small problem | 🞏 | 🞏 |
| Bloody stools | small problem | 🞏 | 🞏 |
| Losing control over your stools | small problem | 🞏 | 🞏 |
| Abdominal/pelvic/rectal pain | small problem | 🞏 | 🞏 |
| Urgency to have a bowel movement | small problem | 🞏 | 🞏 |
|  |  |  |  |

2. Please select the side effect that you think your friend will find most bothersome and the side effect your friend will find least bothersome.

| **Side effect** | **Problem size** | **Most Bothersome (Pick one)** | **Least Bothersome(Pick one)** |
| --- | --- | --- | --- |
| Increased frequency of bowel movements | moderate problem | 🞏 | 🞏 |
| Bloody stools | moderate problem | 🞏 | 🞏 |
| Losing control over your stools | moderate problem | 🞏 | 🞏 |
| Abdominal/pelvic/rectal pain | small problem | 🞏 | 🞏 |
| Urgency to have a bowel movement | moderate problem | 🞏 | 🞏 |
|  |  |  |  |

3. Please select the side effect that you think your friend will find most bothersome and the side effect your friend will find least bothersome.

| **Side effect** | **Problem size** | **Most Bothersome (Pick one)** | **Least Bothersome(Pick one)** |
| --- | --- | --- | --- |
| Increased frequency of bowel movements | big problem | 🞏 | 🞏 |
| Bloody stools | big problem | 🞏 | 🞏 |
| Losing control over your stools | big problem | 🞏 | 🞏 |
| Abdominal/pelvic/rectal pain | small problem | 🞏 | 🞏 |
| Urgency to have a bowel movement | big problem | 🞏 | 🞏 |
|  |  |  |  |

4. Please select the side effect that you think your friend will find most bothersome and the side effect your friend will find least bothersome.

| **Side effect** | **Problem size** | **Most Bothersome (Pick one)** | **Least Bothersome(Pick one)** |
| --- | --- | --- | --- |
| Increased frequency of bowel movements | big problem | 🞏 | 🞏 |
| Bloody stools | moderate problem | 🞏 | 🞏 |
| Losing control over your stools | big problem | 🞏 | 🞏 |
| Abdominal/pelvic/rectal pain | moderate problem | 🞏 | 🞏 |
| Urgency to have a bowel movement | moderate problem | 🞏 | 🞏 |
|  |  |  |  |

5. Please select the side effect that you think your friend will find most bothersome and the side effect your friend will find least bothersome.

| **Side effect** | **Problem size** | **Most Bothersome (Pick one)** | **Least Bothersome(Pick one)** |
| --- | --- | --- | --- |
| Increased frequency of bowel movements | small problem | 🞏 | 🞏 |
| Bloody stools | big problem | 🞏 | 🞏 |
| Losing control over your stools | small problem | 🞏 | 🞏 |
| Abdominal/pelvic/rectal pain | moderate problem | 🞏 | 🞏 |
| Urgency to have a bowel movement | big problem | 🞏 | 🞏 |
|  |  |  |  |

6. Please select the side effect that you think your friend will find most bothersome and the side effect your friend will find least bothersome.

| **Side effect** | **Problem size** | **Most Bothersome (Pick one)** | **Least Bothersome(Pick one)** |
| --- | --- | --- | --- |
| Increased frequency of bowel movements | moderate problem | 🞏 | 🞏 |
| Bloody stools | small problem | 🞏 | 🞏 |
| Losing control over your stools | moderate problem | 🞏 | 🞏 |
| Abdominal/pelvic/rectal pain | moderate problem | 🞏 | 🞏 |
| Urgency to have a bowel movement | small problem | 🞏 | 🞏 |
|  |  |  |  |

7. Please select the side effect that you think your friend will find most bothersome and the side effect your friend will find least bothersome.

| **Side effect** | **Problem size** | **Most Bothersome (Pick one)** | **Least Bothersome(Pick one)** |
| --- | --- | --- | --- |
| Increased frequency of bowel movements | moderate problem | 🞏 | 🞏 |
| Bloody stools | big problem | 🞏 | 🞏 |
| Losing control over your stools | big problem | 🞏 | 🞏 |
| Abdominal/pelvic/rectal pain | big problem | 🞏 | 🞏 |
| Urgency to have a bowel movement | small problem | 🞏 | 🞏 |
|  |  |  |  |

8. Please select the side effect that you think your friend will find most bothersome and the side effect your friend will find least bothersome.

| **Side effect** | **Problem size** | **Most Bothersome (Pick one)** | **Least Bothersome(Pick one)** |
| --- | --- | --- | --- |
| Increased frequency of bowel movements | big problem | 🞏 | 🞏 |
| Bloody stools | small problem | 🞏 | 🞏 |
| Losing control over your stools | small problem | 🞏 | 🞏 |
| Abdominal/pelvic/rectal pain | big problem | 🞏 | 🞏 |
| Urgency to have a bowel movement | moderate problem | 🞏 | 🞏 |
|  |  |  |  |

9. Please select the side effect that you think your friend will find most bothersome and the side effect your friend will find least bothersome.

| **Side effect** | **Problem size** | **Most Bothersome (Pick one)** | **Least Bothersome(Pick one)** |
| --- | --- | --- | --- |
| Increased frequency of bowel movements | small problem | 🞏 | 🞏 |
| Bloody stools | moderate problem | 🞏 | 🞏 |
| Losing control over your stools | moderate problem | 🞏 | 🞏 |
| Abdominal/pelvic/rectal pain | big problem | 🞏 | 🞏 |
| Urgency to have a bowel movement | big problem | 🞏 | 🞏 |
|  |  |  |  |

10. Please select the side effect that you think your friend will find most bothersome and the side effect your friend will find least bothersome.

| **Side effect** | **Problem size** | **Most Bothersome (Pick one)** | **Least Bothersome(Pick one)** |
| --- | --- | --- | --- |
| Increased frequency of bowel movements | moderate problem | 🞏 | 🞏 |
| Bloody stools | moderate problem | 🞏 | 🞏 |
| Losing control over your stools | small problem | 🞏 | 🞏 |
| Abdominal/pelvic/rectal pain | small problem | 🞏 | 🞏 |
| Urgency to have a bowel movement | big problem | 🞏 | 🞏 |
|  |  |  |  |

11. Please select the side effect that you think your friend will find most bothersome and the side effect your friend will find least bothersome.

| **Side effect** | **Problem size** | **Most Bothersome (Pick one)** | **Least Bothersome(Pick one)** |
| --- | --- | --- | --- |
| Increased frequency of bowel movements | big problem | 🞏 | 🞏 |
| Bloody stools | big problem | 🞏 | 🞏 |
| Losing control over your stools | moderate problem | 🞏 | 🞏 |
| Abdominal/pelvic/rectal pain | small problem | 🞏 | 🞏 |
| Urgency to have a bowel movement | small problem | 🞏 | 🞏 |
|  |  |  |  |

12. Please select the side effect that you think your friend will find most bothersome and the side effect your friend will find least bothersome.

| **Side effect** | **Problem size** | **Most Bothersome (Pick one)** | **Least Bothersome(Pick one)** |
| --- | --- | --- | --- |
| Increased frequency of bowel movements | small problem | 🞏 | 🞏 |
| Bloody stools | small problem | 🞏 | 🞏 |
| Losing control over your stools | big problem | 🞏 | 🞏 |
| Abdominal/pelvic/rectal pain | small problem | 🞏 | 🞏 |
| Urgency to have a bowel movement | moderate problem | 🞏 | 🞏 |
|  |  |  |  |

13. Please select the side effect that you think your friend will find most bothersome and the side effect your friend will find least bothersome.

| **Side effect** | **Problem size** | **Most Bothersome (Pick one)** | **Least Bothersome(Pick one)** |
| --- | --- | --- | --- |
| Increased frequency of bowel movements | big problem | 🞏 | 🞏 |
| Bloody stools | small problem | 🞏 | 🞏 |
| Losing control over your stools | moderate problem | 🞏 | 🞏 |
| Abdominal/pelvic/rectal pain | moderate problem | 🞏 | 🞏 |
| Urgency to have a bowel movement | big problem | 🞏 | 🞏 |
|  |  |  |  |

14. Please select the side effect that you think your friend will find most bothersome and the side effect your friend will find least bothersome.

| **Side effect** | **Problem size** | **Most Bothersome (Pick one)** | **Least Bothersome(Pick one)** |
| --- | --- | --- | --- |
| Increased frequency of bowel movements | small problem | 🞏 | 🞏 |
| Bloody stools | moderate problem | 🞏 | 🞏 |
| Losing control over your stools | big problem | 🞏 | 🞏 |
| Abdominal/pelvic/rectal pain | moderate problem | 🞏 | 🞏 |
| Urgency to have a bowel movement | small problem | 🞏 | 🞏 |
|  |  |  |  |

15. Please select the side effect that you think your friend will find most bothersome and the side effect your friend will find least bothersome.

| **Side effect** | **Problem size** | **Most Bothersome (Pick one)** | **Least Bothersome(Pick one)** |
| --- | --- | --- | --- |
| Increased frequency of bowel movements | moderate problem | 🞏 | 🞏 |
| Bloody stools | big problem | 🞏 | 🞏 |
| Losing control over your stools | small problem | 🞏 | 🞏 |
| Abdominal/pelvic/rectal pain | moderate problem | 🞏 | 🞏 |
| Urgency to have a bowel movement | moderate problem | 🞏 | 🞏 |
|  |  |  |  |

16. Please select the side effect that you think your friend will find most bothersome and the side effect your friend will find least bothersome.

| **Side effect** | **Problem size** | **Most Bothersome (Pick one)** | **Least Bothersome(Pick one)** |
| --- | --- | --- | --- |
| Increased frequency of bowel movements | small problem | 🞏 | 🞏 |
| Bloody stools | big problem | 🞏 | 🞏 |
| Losing control over your stools | moderate problem | 🞏 | 🞏 |
| Abdominal/pelvic/rectal pain | big problem | 🞏 | 🞏 |
| Urgency to have a bowel movement | moderate problem | 🞏 | 🞏 |
|  |  |  |  |

17. Please select the side effect that you think your friend will find most bothersome and the side effect your friend will find least bothersome.

| **Side effect** | **Problem size** | **Most Bothersome (Pick one)** | **Least Bothersome(Pick one)** |
| --- | --- | --- | --- |
| Increased frequency of bowel movements | moderate problem | 🞏 | 🞏 |
| Bloody stools | small problem | 🞏 | 🞏 |
| Losing control over your stools | big problem | 🞏 | 🞏 |
| Abdominal/pelvic/rectal pain | big problem | 🞏 | 🞏 |
| Urgency to have a bowel movement | big problem | 🞏 | 🞏 |
|  |  |  |  |

18. Please select the side effect that you think your friend will find most bothersome and the side effect your friend will find least bothersome.

| **Symptom** | **Problem size** | **Most Bothersome (Pick one)** | **Least Bothersome(Pick one)** |
| --- | --- | --- | --- |
| Increased frequency of bowel movements | big problem | 🞏 | 🞏 |
| Bloody stools | moderate problem | 🞏 | 🞏 |
| Losing control over your stools | small problem | 🞏 | 🞏 |
| Abdominal/pelvic/rectal pain | big problem | 🞏 | 🞏 |
| Urgency to have a bowel movement | small problem | 🞏 | 🞏 |
|  |  |  |  |
